# Twenty-four-month clinical performance of different universal adhesives in etch-and-rinse, selective etching and self-etch application modes in NCCL – a randomized controlled clinical trial

**DOI:** 10.1590/1678-7757-2018-0358

**Published:** 2019-04-11

**Authors:** Fatma Dilsad OZ, Esra ERGIN, Simge CANATAN

**Affiliations:** 1 Department of Restorative Dentistry, School of Dentistry, Hacettepe University, Ankara, Turkey.

**Keywords:** Dental adhesives, Dental acid etching, Composite resins, Enamel

## Abstract

**Objective:**

The aim of this randomized, controlled, prospective clinical trial was to evaluate the performances of two different universal adhesives and one etch-rinse adhesive for restoration of non-carious cervical lesions (NCCLs).

**Material and Methods:**

Twenty patients with at least seven NCCLs were enrolled. Lesions were divided into seven groups according to adhesive systems and application modes: GSE: GLUMA Universal-self-etch, GSL: GLUMA Universal-selective etching, GER: GLUMA Universal-etch-and-rinse, ASE: All-Bond Universal-self-etch, ASL: All-Bond Universal-selective etching, AER: All-Bond Universal-etch-and-rinse, SBE (Control): Single Bond2-etch-and-rinse. A total of 155 NCCLs were restored with a nano hybrid composite (Tetric N-Ceram). Restorations were scored with regard to retention, marginal discoloration, marginal adaptation, recurrent caries and post-operative sensitivity using modified United States Public Health Service (USPHS) criteria after one week, 6, 12 and 24 months. Statistical evaluations were performed using Chi-square tests (p=0.05).

**Results:**

The recall rate was 81.9% after the 24-month follow-up. The cumulative retention rates for self-etch groups (GSE: 72.2%, ASE:75%) were significantly lower than other experimental groups (GSL: 93.7%, GER: 100%, ASL: 94.1%, AER: 100%, SBE: 100%) at the 24-month follow-up (p<0.05). Regarding marginal adaptation and marginal discoloration, GSE and ASE groups demonstrated more bravo scores after 6 and 12-month observations but differences were not significant (p>0.05). Only one restoration from ASL group demonstrated post-operative sensitivity at 6 and 12-month observations. No secondary caries was observed on the restorations at any recall. At the end of 24-month observations, no significant differences were detected among groups regarding any of the criteria assessed, except retention.

**Conclusion:**

GLUMA Universal and All-Bond Universal showed better results in etch-and-rinse and selective etching mode compared to the self-etch mode regarding retention. Etch-and-rinse and selective etching application modes of the current universal adhesives tended to provide better clinical outcomes considering the criteria evaluated at the end of 24-month evaluation.

## Introduction

Despite many patients following oral-hygiene instructions carefully, non-carious cervical lesions (NCCLs) have increased among patients in various age populations.^1^ NCCLs are typically seen on the gingival third of the tooth, where the enamel is thinner and the enamel–dentin bond is weaker than in other regions, facilitating substance loss via erosion abrasion and abfraction.^2^ Treatment of NCCLs is necessary because thermal and pH changes can cause severe hypersensitivity.^3^ Various treatments are used to manage NCCLs, including surface-modifying agents and toothpastes that occlude dentinal tubules on superficial lesions. For moderate and deep lesions, the only option to eliminate the clinical symptoms and prevent further loss of tooth structure is restoration, usually.

Composite resin systems are often used for NCCL restoration because, due to their adhesion mechanism, they can be applied directly to these lesions without the need for retentive cavity preparation.^4^ On the other hand, NCCLs typically consist of sclerotic dentin, which can prevent maximum adhesion due to its acid-resistant nature. Nevertheless, self-etch adhesives may not be suitable for highly sclerotic surfaces.^5^ Despite the negative effects of etch-and-rinse adhesive systems, including technical sensitivity and a greater number of steps, they appear to be more reliable than self-etch adhesives.^6^ ^,^ ^7^ However, self-etch adhesives are being widely adopted, as they are more user-friendly, have a reduced number of steps and eliminate the need to use phosphoric acid.^8^ Nevertheless, clinical trials have indicated that self-etch adhesives have higher rates of marginal discoloration than etch-and-rinse systems and negatively influence the aesthetic appearance of restorations.^7^ ^,^ ^9^ In addition, the relatively low enamel bonding strength of self-etch adhesives remains a problem; selective etching of enamel has been suggested as an option to overcome the poor enamel bond strength of self-etch adhesives and improve their clinical success.

Over the past decade, universal adhesives have been introduced to allow clinicians to choose application modes appropriate for a given situation. These adhesives can be used in etch-and-rinse, self-etch, or selective etching modes, thus allowing clinicians to make their own judgment for different cases. Universal adhesives can also provide adhesion to multiple substrates other than tooth surfaces, including resin composites, metals, zirconia, and silica-based ceramics.^10^ The current philosophy of simplifying the application process, saving time, and eliminating errors that may arise from multiple steps, has also led to the manufacturing of universal adhesives. Although these new-generation adhesives are promising, they have been subjected to only a few clinical trials considering their long-term results.^6^ ^,^ ^11^

This randomized, controlled clinical study compared two different universal adhesives in three application modes in restoration of NCCLs after 24 months. The null hypothesis was that there would be no differences among study groups regarding retention, marginal discoloration, marginal adaptation, postoperative sensitivity, and secondary caries based on the United States Public Health Service (USPHS) criteria.

## Material and methods

### Patient selection and study design

This was a randomized, controlled clinical trial. Ethical approval and patient consent forms for the study were reviewed and approved by the Institutional Ethics Committee for Clinical Investigations (Ethic No: KA 17108). Participants were informed about the study protocol and signed a consent form. An experienced clinician recruited participants who met the inclusion criteria among the patients seeking routine dental care from the Restorative Dentistry Department.

Inclusion criteria were: ≥18 years old, no medical or behavioral problems preventing attendance at recall visits; at least seven teeth with NCCLs; and presence of antagonist and adjacent teeth to the teeth to be restored. Exclusion criteria were: poor gingival health; uncontrolled, rampant caries; bruxism; removable partial dentures; and xerostomia. All of the NCCLs selected were of similar size, varying between 1 and 3 mm. NCCLs shallow than 1 mm or deeper than 3 mm were not included in the study. Examinations were carried out with an explorer and periodontal probe to assess the depth and sizes. A cold test was applied for sensitivity examination and patients with severe hypersensitivity were not included. Patients were asked to rate their pain on a scale from 0 to 10 and were excluded if they rated their pain at 7, 8, 9, or 10. Participants were recruited from patients (98 female, 56 male) who visited our department for treatment of their NCCLs.

### Restorative procedures

Patients received dental prophylaxis and oral hygiene instructions 1 week before treatment. Twenty participants with a mean age of 49 years (age range 36–63) received 155 restorations.

All of the NCCLs were restored by the same operator. At least seven lesions were restored at each patient seen, and randomization of different adhesive-system groups was performed using a random number table. Another clinician who was not involved in the research protocol prepared the details of the allocation. The allocation was determined by choosing a number assigned to an adhesive in the tables (only the clinician who was not involved in the study could see these tables). The table consisted of numbers assigned to different groups. Afterwards, all numbers were put in a bag and for each lesion a number was picked from the bag to decide the experimental group. If the patient had more than 7 lesions, numbers were picked again for allocation. This is the reason for the different sample sizes at groups. Each patient did not receive only one group, each patient received 7 different experimental groups. The comparison of the adhesive groups for each category was performed with the Pearson chi-square test. The baseline scores were compared with those at the recall visits using the Cochran’s Q-test followed by McNemar’s test.

**Figure 1:**
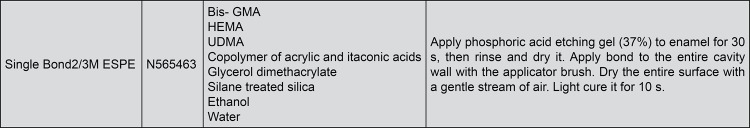
Materials used in the study

UDMA: Urethane dimethacrylate; MDP: 10-Methacryloyloxydecyl dihydrogen phosphate; Bis-GMA: Bisphenol A diglycidyl methacrylate; HEMA: 2-Hydroxyethyl methacrylate

A rubber cup in a slow-speed handpiece was used to clean all lesions before restoring. Afterwards, the lesions were washed and dried, but not desiccated. The NCCLs were isolated by cotton rolls before application. The adhesive and restorative materials were placed according to the manufacturers’ recommendations (Figure 1). All lesions were isolated using cotton rolls before restoring.

A shade selection was carried out for each lesion before applications for proper aesthetic appearance. Then, the composite resin (Tetric N-Ceram; Ivoclar Vivadent, Liechtenstein) was applied according to the manufacturer’s recommendations and light cured for 20 s. The restorations were contoured using flame-shaped fine finishing diamond burs (Diatech, Charleston, SC, USA) in a high-speed handpiece under water spray and polished using OptiDisc dental finishing and polishing discs (Kerr Corporation, Orange, CA, USA).

### Clinical evaluation

Patients were evaluated at baseline, 6, 12, and 24 months after placement. The restorations were checked for retention, marginal adaptation, marginal discoloration, postoperative sensitivity, and secondary caries based on the USPHS criteria by two experienced examiners who were previously calibrated and blinded to the restorative procedures and did not place the restorations. For calibration, 10 photographs that represented each score for each criterion were used. Intraexaminer and interexaminer agreement scores of at least 85% were necessary before beginning the evaluation.

The examiners recorded the data on each material on new and empty evaluation forms, according to tooth numbers, and the forms were not shown to the examiner later to ensure that they were blinded to group assignments at recall visits. The variables retention, marginal adaptation, marginal discoloration, postoperative sensitivity, and secondary caries were evaluated. Scores were ranked as follows: clinically very good (Alpha), clinically sufficient/satisfactory (Bravo), and clinically poor (Charlie). The examiners evaluated all of the restorations independently. In cases in which there was disagreement, the examiners had to reach a consensus before the participant was dismissed.

Restoration retention rates were calculated using the following equation:^11^ ^,^ ^12^ Cumulative failure%=[(PF+NF)/(PF+RR)]×100%. Here, PF is the number of previous failures before the current recall, NF is the number of new failures during the current recall, and RR is the number of restorations recalled for the current recall.

### Statistical analysis

Descriptive statistics were used to present the frequency distributions of the evaluated criteria. The Chi-square test was used to compare the groups at baseline, 6, 12, and 24 months (p=0.05). To distinguish differences in marginal adaptation and marginal discoloration scores within each group by time, further analyses were carried out using the Cochran’s Q test followed by McNemar’s test to compare the data obtained in each evaluation period with baseline (p=0.05).

## Results

In total, 155 restorations of NCCLs were performed in 20 patients (13 female, 7 male) (Table 1). Tooth distributions of restorations according to tooth type and arch are shown in Table 2. Most restorations (65.1%) were at the maxillary arch, at premolars (54.1%). The characteristics of NCCLs are shown in Table 3. Recall rates were 100% at the 6- and 12-month evaluations and 81.9% at the 24-month evaluation.

**Table 1:** Distribution of treated research subjects and non-carious cervical lesions according to sex, age and education level

Characteristics of research aubjects	Number of patients	Number of NCCLs (n%)
**Sex distribution (number of patients)**		
Male	7	57 (36.7)
Female	13	98 (63.2)
Education level		
Primary School	0	-
High School	8	60 (40)
Undergraduate education	10	81 (50)
Graduate education	2	14 (10)
**Age distribution (years)/number of patients**		
20-29	0	-
30-39	3	24 (15.4)
40-49	8	64 (41.2)
50-59	7	52 (33.5)
60-65	2	15 (9.6)

**Table 2:** Distribution of non-cervical caries lesions (NCCLs) according to tooth type and arch

Number of NCCLs	GSE	GSL	GER	ASE	ASL	AER	SBE	Total (n %)
**Arch distribution**								
Maxillary	13	13	13	16	17	14	15	101 (65.1)
Mandibular	8	7	9	4	4	8	14	54 (34.8)
**Tooth distribution**								
Incisors	2	3	2	3	1	7	5	23 (14.8)
Canines	7	3	8	5	3	2	3	32 (20.6)
Premolars	14	11	10	14	12	13	10	84 (54.1)
Molars	2	1	2	1	3	3	4	16 (10.3)

GSE: GLUMA Universal-self-etch; GSL: GLUMA Universal-selective etching; GER: GLUMA Universal-etch-and-rinse; ASE: All-Bond Universal-self-etch; ASL: All-Bond Universal-selective etching; AER: All-Bond Universal-etch-and-rinse; SBE: Single Bond2-etch-and-rinse

**Table 3:** Characteristics of non-cervical caries lesions (NCCLs) included in the study

	Number of NCCLs (n)
**Shape of NCCLs**	
<45	
45-90	40
90-135	93
>135	22
**Cervico-incisal height (mm)**	
<1.5	21
1.5-2.5	41
>2.5-4.0	63
>4.0	30
**Prence in enamel**	
Absent	131
Present	24

### Retention rates

**Figure 2:**
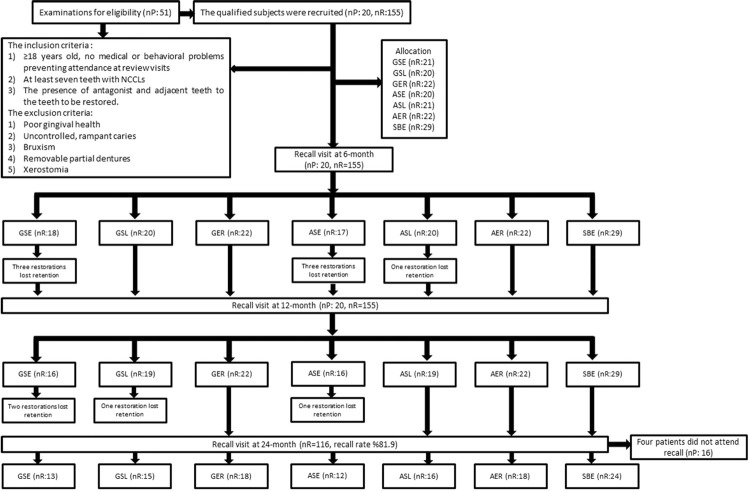
Flowchart of the study. nP: number of patients; nR: number of restorations; GSE: GLUMA Universal-self-etch; GSL: GLUMA Universal-selective etching; GER: GLUMA Universal-etch-and-rinse; ASE: All-Bond Universal-self-etch; ASL: All-Bond Universal-selective etching; AER: All-Bond Universal-etch-and-rinse; SBE: Single Bond2-etch-and-rinse

All of the restorations in the etch-and-rinse groups (GER, AER, and SBE) were scored as Alpha for retention at the 6-, 12-, and 24-month visits (Table 4). During the 24-month evaluation period, one restoration was lost in the ASL group at 6 months (p=0.021), and one in the GSL group at 12 months (p=0.193). In the self-etch groups, the cumulative failure/retention rates of the GSE and ASE groups differed significantly from those of other experimental groups and the baseline values within each group at 6, 12, and 24 months (p=0.001) (Tables 4 and 5) (Figure 2).

**Table 4:** Clinical evaluation outcomes of different adhesive systems

Evaluation criteria	Score				Baseline n (100%)							6-month n (100%)							12-month n (100%)							24-month n (81.9%)			
		GSE	GSL	GER	ASE	ASL	AER	SBE	GSE	GSL	GER	ASE	ASL	AER	SBE	GSE	GSL	GER	ASE	ASL	AER	SBE	GSE	GSL	GER	ASE	ASL	AER	SBE
Retention	Alpha	21	20	22	20	21	22	29	18*	20	22	17*	20	22	29	16*	19	22	16*	19	22	29	13*	15	18	12*	16	18	24
		-100	-100	-100	-100	-100	-100	-100	(85.7)	-100	-100	-85	(95.2)	-100	-100	(76.2)	-95	-100	-80	(95.6)	-100	-100	(72.2)	(93.7)	-100	-75	(94.1)	-100	-100
	Bravo																												
	Charlie								3			3	1			5	1		4	1			5	1		4	1		
									(14.3)			-15	(4.8)			(23.8)	-5		-20	(4.8)			(27.8)	(6.3)		-25	(5.9)		
Marginal adaptation	Alpha	20 (100)	20	22	20	21	22	29	8*	16	18	9*	15	17	23	6*	13*	14*	8*	13*	16*	19*	3*	9*	11*	5*	8*	11*	12*
			-100	-100	-100	-100	-100	-100	(44.4)	-80	(81.8)	(52.9)	-75	(77.3)	(79.3)	(37.5)	(68.4)	(63.6)	-50	-65	(72.7)	(65.5)	(23.1)	-60	(61.1)	(41.7)	-50	(61.1)	-50
	Bravo								10	4	4	8	5	5	6	10	6	8	8	7	6	10	10	6	7	7	8	7	12
									(55.6)	-20	(18.2)	(47.1)	-25	(22.7)	(20.7)	(62.5)	(31.6)	(36.4)	-50	-35	(27.3)	(34.5)	(76.9)	-40	(38.9)	(58.3)	-50	(38.9)	-50
	Charlie																												
Marginal discoloration	Alpha	20 (100)	20	22	20	21	22	29	9*	15	18	8*	15	17	22	6*	14*	14*	7*	12*	13*	18*	4*	8*	11*	5*	7*	9*	12*
			-100	-100	-100	-100	-100	-100	-50	-75	(81.8)	(47.1)	-75	(77.3)	(75.9)	(37.5)	(73.7)	(63.6)	(43.8)	-60	(59.1)	(62.1)	(30.8)	(53.3)	(61.1)	(41.7)	(43.8)	-50	-50
	Bravo								9	5	4	9	5	5	7	10	5	8	9	8	9	11	9	7	7	7	9	9	12
									-50	-25	(18.2)	(52.9)	-25	(22.7)	(24.1)	(62.5)	(26.3)	(36.4)	(56.3)	-40	(40.9)	(37.9)	(69.2)	(46.7)	(38.9)	(58.3)	(56.3)	-50	-50
	Charlie																												
Postoperative sensitivity	Alpha	20 (100)	20	22	20	21	22	29	18	20	22	17	19	22	29	16	19	22	16	19	22	29	13	15	18	12	16	18	24
			-100	-100	-100	-100	-100	-100	-100	-100	-100	-100	-95	-100	-100	-100	-100	-100	-100	-95	-100	-100	-100	-100	-100	-100	-100	-100	-100
	Bravo												1							1									
	Charlie																												
Secondary caries	Alpha	20 (100)	20	22	20	21	22	29	18	20	22	17	20	22	29	16	19	22	16	20	22	29	13	15	18	12	16	18	24
			-100	-100	-100	-100	-100	-100	-100	-100	-100	-100	-100	-100	-100	-100	-100	-100	-100	-100	-100	-100	-100	-100	-100	-100	-100	-100	-100
	Bravo																												
	Charlie																												

(*) indicates significant difference within each group when compared to baseline (p<0.05), GSE: Gluma Universal- self-etch, GSL: GLUMA Universal-selective etching, GER: GLUMA Universal-etch-and-rinse, ASE: All-Bond Universal-self-etch, ASL: All-Bond Universal-selective etching, AER: All-Bond Universal-etch-and-rinse, SBE: Single Bond2-etch-and-rinse

**Table 5:** Relative cumulative frequencies (%) of lost restorations during 24-month follow-up

	6-month	12-month	24-month
GSE	14.3^a*^	23.8^a*^	27.8^a*^
GSL	0.0^b^	5^b^	6.3^b^
GER	0.0^b^	0.0^b^	0.0^b^
ASE	15.0^a*^	20^a*^	25^a*^
ASL	4.8^b^	4.8^b^	5.9^b^
AER	0.0^b^	0.0^b^	0.0^b^
SBE	0.0^b^	0.0^b^	0.0^b^

(*) indicates significant difference within each group when compared to baseline

Different superscripts show significant differences at columns (p<0.05)

### Marginal adaptation

With regard to marginal adaptation, the GSE and ASE groups tended to receive more Bravo scores at 6 (55.6%, 47.1%), 12 (62.5%, 50%), and 24 months (76.9%, 58.3) than the other groups. However, the differences among the groups were not statistically significant at any evaluation (p>0.05). McNemar’s test showed significant changes in marginal adaptation in the ASE and GSE groups at 6 months compared to baseline (p=0.001). The remaining groups began to exhibit significant changes regarding marginal adaptation at 12 months (p=0.001).

### Marginal discoloration

Similar to the marginal adaptation evaluations, Bravo scores indicating moderate marginal discoloration were observed mostly in the ASE and GSE groups at 6, 12, and 24 months, with no significant differences among groups (p=0.098, p=0.350, p=0.767, respectively). On the other hand, the marginal discoloration reached significance in the ASE and GSE groups beginning at 6 months, whereas the other groups showed significant deviations from baseline beginning at 12 months according to Cochran’s Q test following McNemar’s test (p=0.001).

### Postoperative sensitivity

At the 6- and 12-month evaluations, only one restoration, in the ASL group, exhibited postoperative sensitivity (p=0.670, p=0.618, respectively).

### Secondary caries

No secondary caries were found in any of the restorations during the 24-month evaluation period.

## Discussion

In this study, when universal adhesives with self-etch mode were applied, significant clinical failures began to appear at 6 months, and continued to appear at 12 and 24 months (p<0.05). Although the GSE and ASE techniques tended to result in more Bravo scores for marginal adaptation and marginal discoloration at 6, 12, and 24 months, there were no significant differences in any of the criteria evaluated except retention. Therefore, the null hypothesis was partially rejected.

Self-etch adhesives have simplified application techniques compared to etch-and-rinse methods and can be divided into subgroups: two-step self-etch and one-step self-etch. These adhesives require fewer steps, are less sensitive to technical problems, and eliminate the need for application of phosphoric acid and rinsing.^8^ Adhesive systems have evolved from two-bottle to one-bottle etch-and-rinse and self-etch systems. One-step self-etch adhesives act as permeable membranes following photopolymerization, allowing water diffusion to the adhesive surface.^13^ This may be associated with the inferior survival rates of the self-etch adhesives clinically. In addition, no surface etching procedures, especially at the enamel, are considered to result in lower retention rates of self-etch adhesives.^7^ ^,^ ^9^

Studies of etch-and-rinse adhesives found superior clinical performance compared to simplified adhesives.^7^ ^,^ ^14^ However, clinical application times and numbers of steps in application are not user-friendly. Van Meerbeek, et al.^15^ (2011) reported that etching enamel with phosphoric acid formed sufficient sealing and the bonding interface was protected against degradation. A long-term study of NCCLs indicated that the etching step affected the clinical performance of restorations.^16^ A systematic review evaluated average failure rates of adhesive systems annually and pointed out that three-step etch-and-rinse and two-step self-etch adhesives were the most effective at NCCLs, with annual failure rates of 4.8% and 4.7%, respectively. The highest annual failure rate (8.1%) was observed at simplified one-step self-etch adhesives. Any simplification of the clinical procedures resulted in loss of bonding effectiveness due to hydrolysis and elution of interface components.^17^

The provisional acceptance criteria for enamel and dentin adhesives according to the American Dental Association (ADA) are maximal 5% restoration loss or microleakage at a 6-month recall.^11^ ^,^ ^12^ In addition, the cumulative incidence of clinical failures should be tested in two independent clinical studies, and failure rates at 18 months must be lower than 10% for retention and microleakage. In this study, GLUMA Universal and All-Bond Universal adhesives had rates of retention loss in self-etch mode higher than the rates considered acceptable by the ADA. Similar to our findings, Brackett, et al.^18^ (2002) evaluated the clinical performance of a self-etch adhesive in Class V lesions, and reported 24% retention loss at 6 months and 35% at 12 moths, rates that were considered unacceptable based on the ADA criteria. On the other hand, Van Dijken^19^ (2004) reported 3.9% retention loss for a self-etch adhesive system and 2.2% retention loss for an etch-and-rinse system at 6 months. Similar to this investigation, in another study, restorations placed using a self-etch adhesive had 93% survival at 18 months.^20^

Universal adhesives have been developed from self-etch adhesives and are referred to as “multimode” because they can be used in self-etch, selective etch, and etch-and-rinse modes on various adherent substrates, including enamel, dentin, metal alloys, and ceramics.^21^ ^,^ ^22^ Laboratory studies that evaluated the bond strength of universal adhesives using the etch-and-rinse and self-etch modes emphasized that bond strength on enamel was significantly better in the etch-and-rinse mode.^23^ ^,^ ^24^ However, some studies^22^ ^,^ ^25^ have reported no differences in bond strength between etched dentin and self-etched dentin when universal adhesives were used.

Commercially available universal adhesives usually contain 10-methacryloyloxydecyl dihydrogen phosphate (MDP). This functional monomer can form a salt with the calcium in hydroxyapatite using a polymerizable methacrylate group. MDP is also a hydrophobic molecule and therefore has the ability to decrease water permeability.^26^ The use of a universal adhesive containing MDP (Scotchbond Universal) in different modes was evaluated in a clinical trial; in self-etch mode, the adhesive resulted in 6% retention loss (three restorations) at 6 months and no significant differences were observed in comparison to an etch-and-rinse group.^7^ However, the present study found significant differences between self-etch and etch-and-rinse groups at 6 months. Both universal adhesives in this study contained MDP monomer, and at the 24-month evaluations they showed 27.8% (GSE) and 25% (ASE) retention loss in self-etch mode, whereas none of the restorations in the etch-and-rinse groups was lost.

Water and HEMA have the ability to expand dried and collapsed dentin, also increasing water content and expansion.^27^ Although the All-Bond Universal groups in this study contained water/HEMA that expanded collagen in dentin in the selective etching and self-etch modes, the ASE group had significantly higher failure rates compared to the ASL and AER groups. It should be considered that the type of solvent also affects the degree of moisture of dentin in clinical practice. GLUMA Universal contains acetone as a solvent, whereas All-Bond Universal and Single Bond2 contain ethanol. The evaporation behaviors of the solvents might affect the long-term results of the adhesives used since acetone and ethanol have different boiling temperatures and vapor pressures. Degradation of the adhesive layer is likely to be higher at acetone-based adhesive systems compared to ethanol-based systems after evaporation. In addition, thin layers of adhesives are more sensitive to inhibition of polymerization by oxygen.^28^ One clinical trial reported that an acetone-based adhesive system had a lower retention rate than an ethanol-based adhesive system at 36 months, and did not meet the ADA requirement for an acceptable failure rate.^7^ ^,^ ^29^ However, this study showed no differences between ethanol- and acetone-based universal adhesives in any of the criteria examined during the 24-month evaluation.

The acidity of adhesives also differs, which may affect clinical performance. An *in vitro* study^30^ investigated a self-etch adhesive with a mild pH in different modes (etch-and-rinse and self-etch) and found that the self-etch mode results were inferior to those of the etch-and-rinse mode. Conversely, a systematic review^31^ of the clinical performance of adhesives found that mild two-step self-etch adhesives resulted in the best clinical bonding effectiveness in NCCLs, whereas the results for strong self-etch (pH<1.5) and two-step etch-and-rinse adhesives were unsatisfactory. One long-term clinical study compared a strong self-etch adhesive to a mild self-etch adhesive and concluded that there was no significant difference in their performance at 6 years. The present study found no difference between mild (GLUMA Universal) and ultra-mild (All-Bond Universal) universal adhesives used in self-etch mode.

An important finding of this investigation was that the rates of marginal discoloration were especially high in restorations placed using universal adhesives in self-etch mode, which was attributed to the lower bonding ability of self-etch adhesives to unetched enamel than to etched enamel.^32^ In addition, the methacrylate copolymers in the resin composites are hydrophilic, which causes water sorption from the oral environment when exposed externally to salivary fluids and internally to underlying hydrated dentin.^33^ The swelling of polymer reduces friction between chains, negatively affecting mechanical properties.^34^ This not only causes marginal discoloration but also affects the marginal adaptation of restorations. However, in the present study this correlation did not lead to gap formation or secondary caries. Marginal discoloration has been reported to appear over time in many studies,^7^ ^,^ ^35^ ^,^ ^36^ but can generally be resolved easily by repolishing.

To our knowledge, few studies have clinically evaluated universal adhesives and there are no published data comparing the clinical performance of different universal adhesives. Loguercio, et al.^37^ (2015) compared different application modes of Single Bond Universal and reported that when the universal adhesive was applied in self-etch mode, significant signs of degradation were evident at 36 months with regard to marginal adaptation and marginal discoloration. Although the evaluation period in their study was longer than in the present study, their results are consistent with our findings. In contrast, Lawson, et al.^6^ (2015) reported that Single Bond Universal showed similar results in both etch-and-rinse and self-etch modes at a 24-month evaluation. The retention rates for self-etch mode at 36 (89%) and 24 months (94.9) were very high compared to the present study. Although All-Bond Universal is an ethanol-based MDP-containing adhesive similar to Single Bond Universal, this study found a 75% retention rate at 24 months in self-etch mode. The differences may be related to the clinicians who performed the restorations, patient factors, properties of the NCCLs, numbers of restorations in the experimental groups, and study design.

Considering the failure distributions of the restorations according to upper or lower arch; in the GSE group, of the 8 lower teeth restored, 4 (50%) failed, and in the ASE group, of the 4 lower teeth, there was 1 (25%) failure. As for the upper teeth that failed, in GSE, of the 13 treated teeth, only 3 (23%) failed, and in the ASE group, of the 16 upper teeth only 1 (6%) failed. Although no statistically significant correlation could be detected, it can be speculated that the restorations in the lower arch had higher failure rates in the present study. On the other hand, this finding requires to be validated with the further clinical studies.

As this study evaluated seven groups, all groups had a limited number of restorations that remained within ADA limits (20–29 restorations *per* group). On the other hand, this outcome was due to the purpose of comparing two different universal adhesives and an etch-and-rinse control in a split-mouth study design for the very first time. Additionally, as it is almost impossible to find NCCLs in an exact same depth on split-mouth studies, none of the included lesions was shallower than 1 mm or deeper than 3 mm to eliminate the influence of lesion depth depending on the previous studies.^14^ ^,^ ^35^ Still, the influence of depth variations within 1-3 mm range on the clinical performance of NCCL restorations requires to be investigated in further studies, as they still have the potential to affect the performance of such restorations. The cervico-incisal or cervico-occlusal sizes of the NCCLs in this study were categorized into 4 groups. The failure numbers from each group were similar (GSE: 1 failure at <1.5, 2 failures at 1.5-2.5, 2 failures at >2.5-4.0, GSL: 1 failure at <1.5, ASE: 1 failure at 1.5-2.5, 3 failures at >2.5-4.0, ASL: 1 failure at <1.5), so that no correlation could be detected. Nevertheless, further long-term clinical studies should be reported to demonstrate the significance of sizes in NCCLs.

Another limitation of this investigation was that the dentin sclerosis levels of lesions were not evaluated before restoration as in most previous studies of universal adhesives.^6^ ^,^ ^37^ Studies reported that sclerotic lesions did not show significantly different retention rates^38^ nor marginal discoloration rates^39^ compared to non-sclerotic lesions. On the other hand, a currently published *in vitro* study^40^ reported that the functional monomer in the adhesive might have an effect on adhesive interface depending on the morphology of the substrate which is directly related to the dentin sclerosis level. However, as it is very difficult to extrapolate the results of an *in vitro* study to clinical conditions, these findings must be interpreted carefully and long-term clinical evaluations must be carried out to test these findings so reliable judgements can be made.

## Conclusion

Based on the 24-month results of this randomized controlled clinical trial, it can be concluded that GLUMA Universal and All-Bond Universal adhesives used in self-etch mode demonstrated unacceptable retention rates at 6, 12, and 24 months. Universal adhesives in etch-and-rinse mode and Single Bond2 showed minor differences in terms of marginal adaptation and marginal discoloration at 24 months. The etch-and-rinse and selective-etch application modes of GLUMA Universal and All-Bond Universal adhesives produced acceptable clinical results that were as good as the tested etch-and-rinse adhesive system (Single Bond2) at the 24-month evaluation.
